# Influence of repeated anaesthesia on physiological parameters in male Wistar rats: a telemetric study about isoflurane, ketamine-xylazine and a combination of medetomidine, midazolam and fentanyl

**DOI:** 10.1186/s12917-014-0310-8

**Published:** 2014-12-31

**Authors:** Maike Albrecht, Julia Henke, Sabine Tacke, Michael Markert, Brian Guth

**Affiliations:** Department of Nonclinical Drug Safety, Biological Laboratory Service, Boehringer Ingelheim Pharma GmbH & Co. KG, Birkendorfer Str. 65, 88397 Biberach, Germany; Department of Veterinary Clinical Sciences, Clinic for Small Animals-Surgery, Justus-Liebig University, Frankfurter Str. 108, 35392 Giessen, Germany; Department of Drug Discovery Support, General Pharmacology, Boehringer Ingelheim Pharma GmbH & Co. KG, Birkendorfer Str. 65, 88397 Biberach, Germany

**Keywords:** Rat, Repeated anaesthesia, Isoflurane, Ketamine-xylazine, Medetomidine-midazolam-fentanyl, Telemetry, Heart rate, Blood pressure, Body temperature, Body weight

## Abstract

**Background:**

This study evaluated the influence of repeated anaesthesia using isoflurane (ISO, 2–3 Vol%), ketamine-xylazine (KX, 100 mg·kg^−1^ + 5 mg·kg^−1^, i.m.) or a combination of medetomidine-midazolam-fentanyl (MMF, 0.15 mg·kg^−1^ + 2.0 mg·kg^−1^ + 0.005 mg·kg^−1^, i.m.) on heart rate (HR), arterial blood pressure (BP), body temperature (BT), duration of anaesthetic intervals and body weight (BW) in Wistar rats. Rats were instrumented with a telemetric system for the measurement of systolic, diastolic and mean arterial pressure (SAP, DAP, MAP), pulse pressure (PP), HR and BT during induction, maintenance and recovery of anaesthesia. Each anaesthesia was performed six times within three weeks. KX was not antagonized, but ISO delivery was terminated 40 minutes after induction and MMF was reversed with atipamezole-flumazenil-naloxone (AFN, 0.75 mg·kg^−1^ + 0.2 mg·kg^−1^ + 0.12 mg·kg^−1^, s.c.).

**Results:**

With repeated anaesthesia, ISO showed a decrease of HR and BP. A significant decrease of PP could be observed with repeated anaesthesia using MMF. HR and BP were not affected by repeated KX anaesthesia, but we noted a reduction of sleeping time and BW. Neither MMF nor ISO showed significant differences in the duration of anaesthetic intervals and BW. With KX we observed tissue necrosis at the injection site and surgical tolerance was not achieved in 25% of the anaesthesias performed.

**Conclusion:**

HR, BP values, BT, duration of anaesthetic intervals and BW were affected differently by repeated anaesthesia performed with ISO, KX or MMF. ISO produced a reproducible anaesthesia, thereby being suitable for repeated use, but with a decrease of HR and BP throughout the six anaesthesias. The use of ISO in cases where these parameters should be unaffected is therefore not advised. The inability to produce a surgical tolerance, the reduction of sleeping time and BW, as well as the tissue necrosis are significant contraindications for a repeated use of KX. Only mild changes of BP were found with repeated MMF anaesthesia, so it seems suitable for serial use, unless the high BP and the low HR during the surgical plane of anaesthesia are undesirable for a special procedure.

## Background

Repeated general anaesthesia may be required in animal experiments several times a day, or on different days. This might arise, for example, in longitudinal imaging studies, in pharmacokinetic studies where a serial collection of blood or other samples is required and in studies where surgical procedures or drug administrations are performed under anaesthesia on different days. Also in veterinary practice, repeated anaesthesias may be required for some therapeutic treatments (painful wound care, regular dental care…) [1-4]. The rat is a commonly used laboratory animal and has gained popularity as a pet [5]. For producing general anaesthesia in the rat, isoflurane (ISO), a mixture of ketamine-xylazine (KX) or a combination of medetomidine-midazolam-fentanyl (MMF) have been used [5-8]. In a previous study we have shown the influence of these anaesthesias on HR, SAP, DAP, MAP, PP and BT in adult, male Wistar rats [9]. With ISO we observed a significant increase of HR and a mild hypotension during anaesthesia. A marked hypertension and a significant decrease of HR were seen during MMF anaesthesia. The slightest alterations on the parameters measured were observed with KX, but prolonged monitoring was needed, because of long wake-up and recovery periods. It is not known, however, if the effects observed change with repeated anaesthesia, which may be of importance, since such changes could have an impact on the outcome of studies requiring repeated anaesthesia. Some studies have already evaluated influences of different anaesthetic regimes on the animal’s physiology, but none of these studies used telemetric data assessment to evaluate continuously alterations of HR, BP values and core BT before, during and after anaesthesia. Radiotelemetry is regarded as the state-of-the-art monitoring method to provide reliable values of cardiovascular parameters and body temperature without stressful manipulations of the animal which could affect the data collected [10-14].

The aim of this study was to assess the effects of repeated anaesthesia using ISO, KX or MMF in telemetrically instrumented male Wistar rats on HR, SAP, DAP, MAP, PP, BT and duration of different anaesthetic stages. We hypothesized that change in the parameters measured would be observed with repeated anaesthesia. Further it was our intention to assess BW during the series of performed anaesthesias, because significant weight loss is one critical point in evaluating the general health condition of rodents.

## Methods

### Animals

Nine male Wistar rats with a mean body weight of 275 ± 24 g were obtained from Charles River Laboratories, Sulzfeld, Germany. They were housed in groups of three in a Makrolon® cage (Type IV). The cages contained a wooden bedding material (Lignocel select fine, J. Rettenmaier & Söhne GmbH + Co. KG, Rosenberg, Germany) and as cage enrichment a wooden chewing block and two red, transparent plastic tubes were provided. The cages were changed twice per week. Rats were given free access to a commercially available diet (3438 maintenance diet, KLIBA NAFAG, Provimi Kliba AG, Kaiseraugst, Switzerland) and tap water. The animals were acclimated for two weeks prior to surgical implantation of the radiotelemetry transmitter in an animal room maintained at 22 ± 2°C and 55 ± 10% relative humidity, on a twelve-hour light–dark cycle (beginning at 6:00 am) with at least 15 air changes per hour. A radio was turned on during the working hours to reduce possible stress caused by environmental noises. After the implantation surgery and a rehabilitation period of at least two weeks, rats were used in a previous anaesthetic study, which lasted nine weeks [9]. The rats were then given a recovery period of at least three weeks prior to the start of the present study. At the beginning of the present study, the rats had a mean body weight of 434 ± 48 g.

### Radiotelemetry transmitter implantation surgery

The radiotelemetry transmitter (DSI PhysioTel™ C50-PXT) was implanted under general anaesthesia. Anaesthesia was induced with MMF (same dosage as used in this study) and was maintained with a second injection of MMF (one third of the initial dosage) after 45 minutes. Analgesics and antibiotics were administered prior to surgery: 50 mg·kg^−1^ metamizole i.m. (Novalgin®, 500 mg·ml^−1^, Sanofi Aventis, Frankfurt/Main, Germany), 1 mg·kg^−1^ meloxicam s.c. (Metacam®, 20 mg·ml^−1^, Boehringer Ingelheim, Ingelheim/Rhein, Germany), 10 mg·kg^−1^ enrofloxacin s.c. (Baytril® 2.5% ad us. vet., Bayer, Leverkusen, Germany). After induction of anaesthesia, protective eye lubricant (VitA-POS, Ursapharm, Saarbrücken, Germany) was administered in both eyes, the rat was laid in a supine position in the middle of a water heating pad, shaved on the ventral site and desinfected with Kodan®-spray (Schülke & Mayr, Norderstedt, Germany) and Betaisodona®-solution (Mundipharma GmbH, Limburg (Lahn), Germany). Supplemental Oxygen was provided via a head chamber throughout the anaesthesia. The rat was covered with a sterile foil and an incision was made along the *linea alba* to open the abdominal cavity. The intestines were moved towards the diaphragm and held in position with a wet swab. The aorta was exposed and blood flow was stopped with two vascular clips. The blood pressure catheter was inserted between the two clips and fixed with one drop of tissue glue (Histoacryl®, B.Braun, Aesculap AG, Tuttlingen, Germany). The clips were carefully opened and the swab was removed. The transmitter was fixed with a permanent suture (Mersilene® 3–0, Ethicon®, Johnson-Johnson Medical GmbH, Norderstedt, Germany) to the abdominal wall. Before the abdominal cavity was closed with a muscle and skin suture (Vicryl® 3–0, Ethicon®, Johnson-Johnson Medical GmbH, Norderstedt, Germany), the ECG leads were exteriorized through the muscle layer. One ECG lead was placed subcutaneously near the sternum and the other was placed on the ventral site of the trachea, both fixed to the nearby muscle tissue with a non-absorbable suture (Mersilene® 3–0, Ethicon®, Johnson-Johnson Medical GmbH, Norderstedt, Germany). Twenty ml·kg^−1^ warmed lactated ringer’s solution (Ringer-Lactat nach Hartmann B. Braun, B/BRAUN, Melsungen, Germany) were administered subcutaneously and anaesthesia was reversed with a s.c. injection of AFN (same dosage as used in this study). After surgery the rats received analgesics for another two days.

### Experimental design

The following three anaesthetic regimes were performed repeatedly in male Wistar rats: 1) an inhalational anaesthesia with 2–3 Vol% isoflurane (Forene® 100% (V/V), Abbott, Wiesbaden, Germany) at maintenance, terminated after 40 minutes, 2) a combination of 100 mg · kg^−1^ ketamine (Ketavet®, 100 mg·ml^−1^, Pfizer, Berlin, Germany) and 5 mg·kg^−1^ xylazine (Rompun® 2%, 20 mg·ml^−1^, Bayer, Leverkusen, Germany) administered i.m., which was not reversed, and 3) an anaesthesia consisting of 0.15 mg·kg^−1^ medetomidine (Domitor, 1 mg·ml^−1^, Orion Pharma, Espoo, Finland), 2 mg·kg^−1^ midazolam (Dormicum®, 5 mg·ml^−1^, Roche, Grenzach-Wyhlen, Germany) and 0.005 mg·kg^−1^ fentanyl (Fentanyl®-Janssen, 0.05 mg·ml^−1^, Janssen, Wien, Austria), which was administered i.m. and antagonized after 40 minutes with a s.c. injection of the antagonists 0.75 mg·kg^−1^ atipamezole (Antisedan®, 5 mg·ml^−1^, Orion Pharma, Espoo, Finland), 0.2 mg·kg^−1^ flumazenil (Flumazenil Hexal®, 0.1 mg·ml^−1^, Hexal, Holzkirchen, Germany) and 0.12 mg·kg^−1^ naloxone ( Naloxon Inresa, 0.4 mg·ml^−1^ l, Inresa, Freiburg, Germany). Anaesthesias were performed twice weekly (Monday + Thursday or Tuesday + Friday) over three consecutive weeks. The six anaesthesias were described in the following text as *run 1* to *run 6*. The anaesthetic regimes were assigned randomly to each individual rat. Each rat received only two of the three anaesthetic treatments for six times with a wash out and recovery period of at least two weeks between them. SAP, DAP, MAP, PP, HR and BT were measured continuously before, during and after each anaesthesia. To monitor the anaesthetic depth, the righting reflex and the pedal withdrawal reflex on the fore- and hind limbs were measured (classified with +, ±, and -) after 2.5, 5, 7.5, 10, 15, 20, 30, 40, 42.5, 45, 47.5, 50, 60, 70…minutes until the righting reflex had returned. One *run* was divided into different intervals: 1) an acclimatization period prior to the anaesthesia of at least two hours for assessing baseline values, 2) the performance of anaesthesia, and 3) a recovery period. To analyse alterations in the duration of the anaesthetic effect, the anaesthesia period was further divided in different anaesthetic stages: 1) **induction time** (defined as the time from application to loss of the righting reflex), 2**) time of non-surgical tolerance** (defined as the time from loss of the righting reflex until loss of all reflexes tested), 3) **time of surgical-tolerance** (defined as the time from loss of all reflexes tested until regaining of at least one reflex), 4) **wake-up period** (defined as the time from regaining the first reflex until regaining the righting reflex) , and 5) **recovery period** (defined as the time from regaining the righting reflex until measurement was terminated). The end of measurement was not earlier than six hours after induction. Sleeping times longer than six hours occurred with KX, therefore data assessment was prolonged and terminated not earlier than two hours after the righting reflex had returned. Additionally, each rat was weighed to calculate the individual dose of injectable anaesthetics and to assess a possible influence of repeated anaesthesia on BW. Daily monitoring of the animals’ general condition and well-being were carried out, especially with regard to inflammation and tissue necrosis at the hind legs after injection of anaesthetics. Only visible changes (open wounds, lameness) were assessed, because the animals were used in further studies.

The Animal Care and Ethics Committee of the Regional Authority in Tuebingen, Baden-Wuerttemberg, Germany approved this study (Approval number: 12–038).

### Procedure

The experimental set-up was the same as that used in the previous study [9]. To reduce variability, anaesthesias were performed by the same veterinarian supported always by the same animal care takers, who were familiar with the animals. To ensure a thorough monitoring of each rat during anaesthesia, not more than three rats were anaesthetized per day. The measurements have been carried out in the same laboratory as described below:

The implanted radiotelemetry transmitters of the three rats were switched on using a magnet before starting the measurements. The rats were then placed individually into a Makrolon® cage, each positioned directly on a receiver plate, and continuous data collection of arterial blood pressure and BT was started. The cages contained bedding material (Lignocel select fine, J. Rettenmaier & Söhne GmbH + Co. KG, Rosenberg, Germany), a red, transparent plastic tube and water was provided in a water bottle during the entire measurement. Following recommendations to pre-warm animals prior to anaesthetic induction, a warm water heating pad was placed between the receiver plate and the Makrolon® cage and it was maintained at 38°C throughout the measurement period [15,16]. To facilitate reaching resting baseline values, rats were housed individually, food was withdrawn, each cage was covered with a cloth, a radio was switched on (because rats were habituated to background music), and the operator left the room for at least two hours. After this acclimatization period the operator started with the anaesthetic induction of the first rat, while the other two rats remained in their covered cages. Performing anaesthesia on one rat did not appear to have an influence on the measured parameters of the other two. All anaesthesias were carried out close to the receiver plate to ensure the continuous capture of the telemetric signal and are described in detail below:

**ISO**: An anaesthetic chamber was prefilled with 5 Vol% ISO supplemented with oxygen. The rat was placed in the whole body chamber, which was positioned directly on the receiver plate. Loss of righting reflex was tested by tipping over the anaesthetic chamber. When the righting reflex was lost, the animal was laid on its back on the receiver plate and ISO was administered using a nose cone. The concentration of ISO was reduced to 2–3 Vol% to maintain an anaesthetic depth of surgical tolerance. ISO administration was terminated 40 minutes after induction of anaesthesia and the rat was laid back in its cage, which was placed on the receiver plate again. The rat was positioned in a dorsal recumbency to test reflexes until regaining the righting reflex.

**KX**: Ketamine (100 mg·kg^−1^) and xylazine (5 mg·kg^−1^) were mixed in one syringe with a total volume of 1.25 ml·kg^−1^, which is too much to be injected all at once. Therefore, half of this volume was administered in the caudal part of the thigh muscle of each hind leg. As soon as the righting reflex was lost, the rat was placed directly on the receiver plate in dorsal recumbency. Oxygen was supplemented using a head chamber. Rats received no treatment to reverse the anaesthesia. Therefore, the duration of the sleeping time was variable. Accordingly, rats were laid on their backs on the receiver plate until the righting reflex returned and after that they were put back in their cage for the rest of the measurements. Wake-up and recovery periods lasted several hours and therefore prevented a normal food and water uptake. Therefore, each rat was administered s.c. 5 ml of warmed lactated ringer’s solution (Ringer-Lactat nach Hartmann B. Braun, B. Braun, Melsungen, Germany) one hour after induction of anaesthesia.

**MMF**: 0.15 mg·kg^−1^ medetomidine, 2 mg·kg^−1^ midazolam and 0.005 mg·kg^−1^ fentanyl were combined in one syringe with a total volume of 0.65 ml · kg^−1^. The mixture was administered i.m. in the caudal part of the thigh muscle of one hind leg while holding the rat next to the receiver plate. The rat was put back in its cage until loss of the righting reflex. The rat was placed directly on the receiver plate in dorsal recumbency and oxygen was provided using a head chamber. A mixture of antagonists (0.75 mg·kg^−1^ atipamezole + 0.2 mg·kg^−1^ flumazenil + 0.12 mg·kg^−1^ naloxone) was administered s.c. 40 minutes after induction of the anaesthesia and the rat was placed in dorsal recumbency in its cage to determine the time when the righting reflex was regained.

### Statistical analysis

For the continuous telemetric data acquisition the software NOTOCORD-hem™ was used and the raw data were further evaluated with MS Excel with subsequent export to SAS 9.3 (SAS Institute Inc., Cary, North Carolina, USA) for the statistical evaluation. The statistical analysis was performed separately for each treatment group (ISO, KX, MMF), parameter (SAP, DAP, MAP, PP, HR, BT, BW) and anaesthetic interval (induction time, time of non-surgical tolerance, time of surgical tolerance, wake-up period, recovery period). The standardized area under the curve (AUC divided by interval length) was calculated for each animal individually using the trapezoidal rule for each anaesthetic interval. Baseline values were calculated for each animal as the standardized AUC including the measurements from 60 to 10 minutes before induction of anaesthesia. The standardized AUC was then used as target variable for the statistical evaluation. The differences between the *1st run* and its consecutive *runs* were analysed. An analysis of covariance (ANCOVA) for repeated measurements was calculated including baseline as covariate. The parameter body weight was analysed by an analysis of variance (ANOVA). In addition, the length of the intervals was analysed using an ANOVA model for repeated measurements. To exclude effects of repeated anaesthesia on baseline values, parameters were analysed during the baseline interval using an analysis of variance (ANOVA). Treatment effects for the telemetry parameters were quantified by mean differences, based on the adjusted mean values and their two-sided 95% confidence interval. Treatment effects for body weight and the length of the intervals were quantified by the differences in mean and their two-sided 95% confidence interval. The level of significance was fixed at α = 5%. A p-value of less than 0.05 was considered to be statistically significant.

## Results

During the course of this study we had four runs that needed to be excluded from the analysis. One rat anaesthetized with ISO in *run 6* developed a poor blood pressure signal and could not be evaluated. Another rat died during induction of KX anaesthesia in *run 6*. With MMF there was one rat in its *6th run* with an elevated BT and an increased HR already during the baseline assessment such that we excluded that rat from the evaluation. Finally, one rat had a failed application of MMF in its *4th run* due to strenuous defensive movements of the animal. Therefore, these four measurements were excluded from the statistical analysis of HR, BP values, BT and duration of anaesthesia, but we still included these rats for the statistical analysis of BW.

Our intention to divide the anaesthesias in five different anaesthetic stages failed for KX, because in 25% of the anaesthesia runs with KX the animals did not reach a stage of surgical tolerance. Instead of excluding these measurements from the statistical analysis, we decided to divide all KX anaesthesias (irrespective of whether or not surgical tolerance was reached) only in three different anaesthetic stages: 1) an **induction time** (time from injection until loss of the righting reflex), 2) a **time of non-surgical tolerance** (time from loss of the righting reflex until regaining the reflex) and 3) a **recovery period** (time from regaining the righting reflex until end of measurement).

### Duration of anaesthetic stages

**Figure 1:**
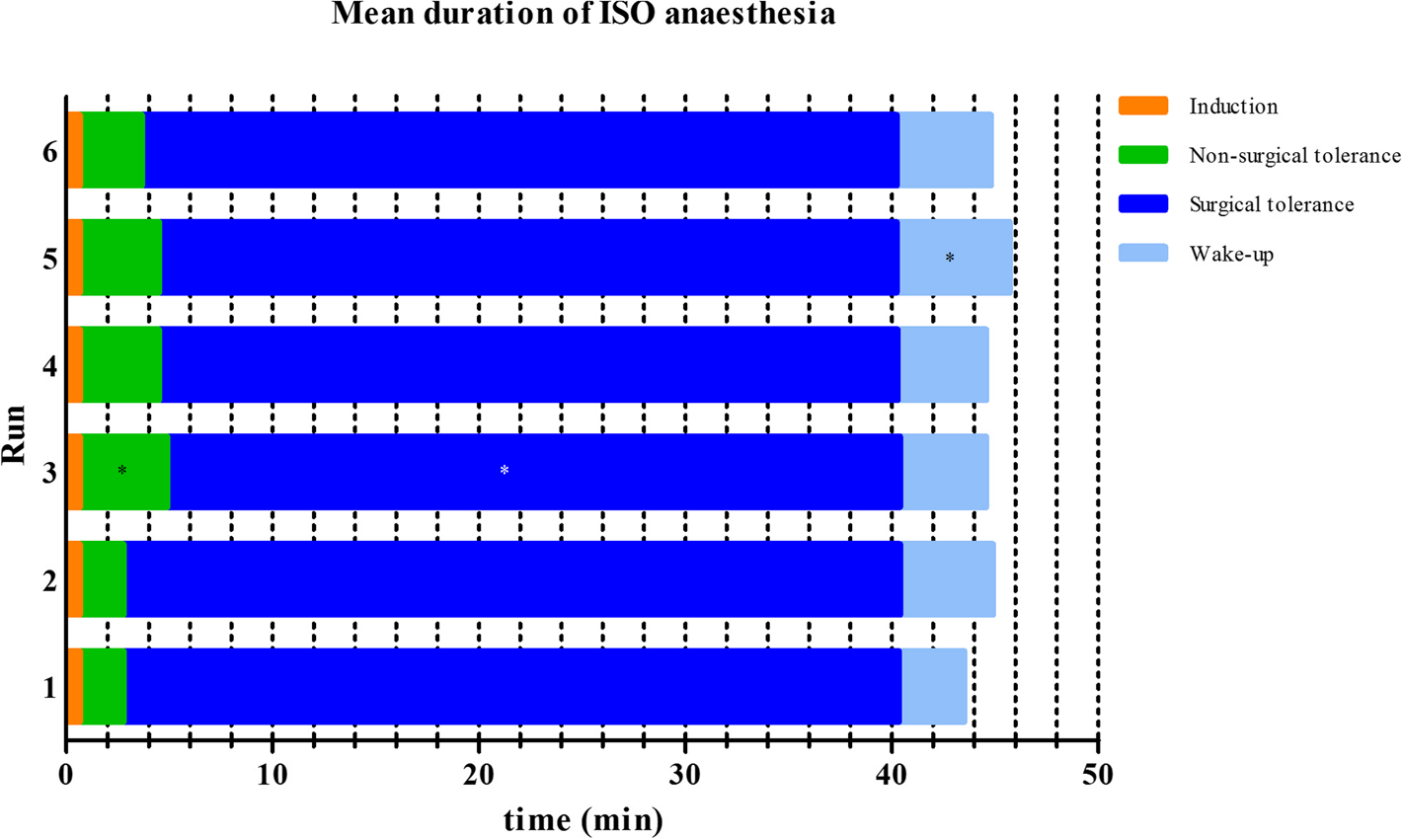
**Mean duration of ISO anaesthesia.** Duration of anaesthesia was divided in four different anaesthetic stages. *Statistical significant difference (p value < 0.05) based on the ANOVA for the comparison of the anaesthetic stages of *run 2–6* with the anaesthetic stage of *run 1*.

**Figure 2:**
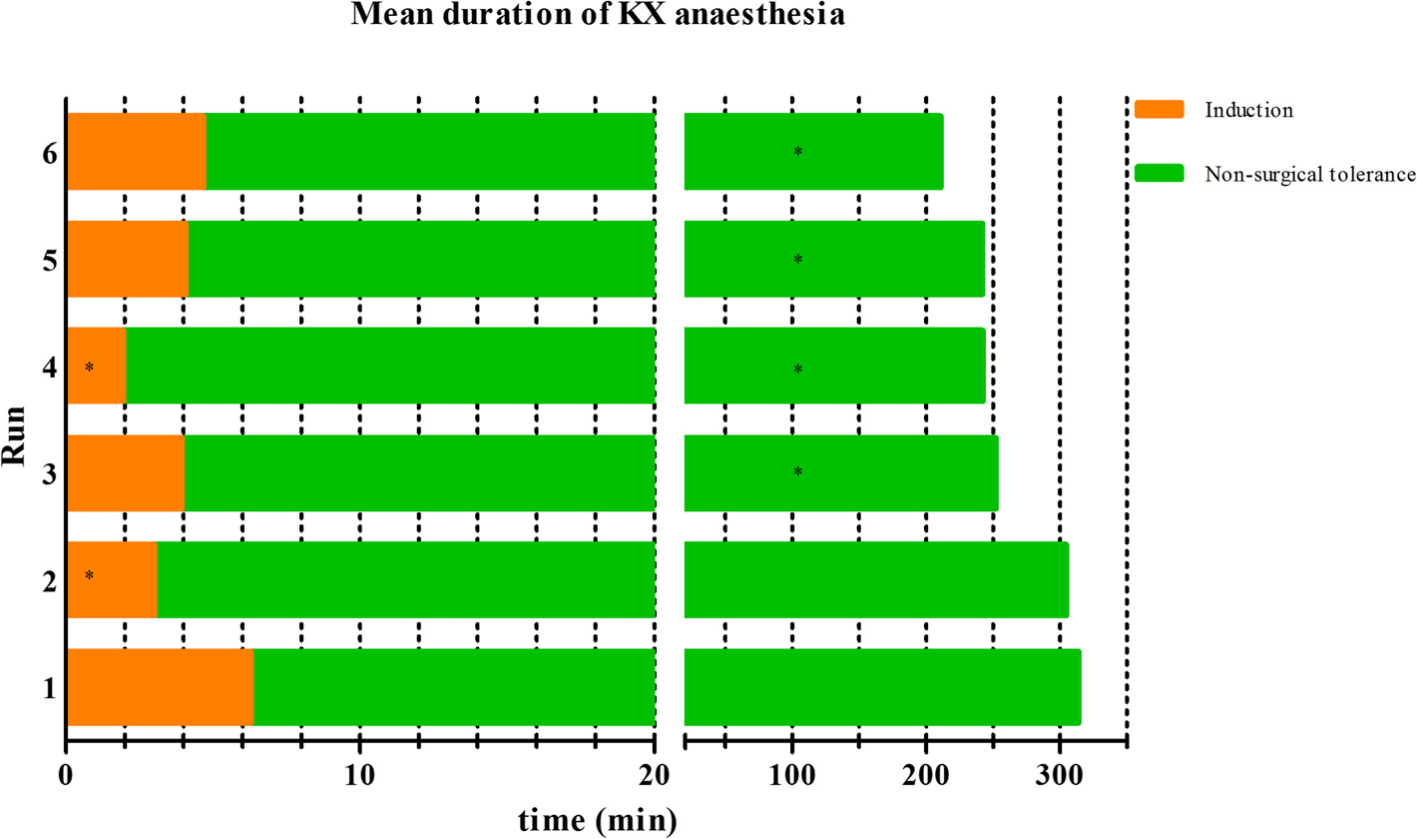
**Mean duration of KX anaesthesia.** Duration of anaesthesia was divided in two different anaesthetic stages. *Statistical significant difference (p value < 0.05) based on the ANOVA for the comparison of the anaesthetic stages of *run 2–6* with the anaesthetic stage of *run 1*.

**Figure 3:**
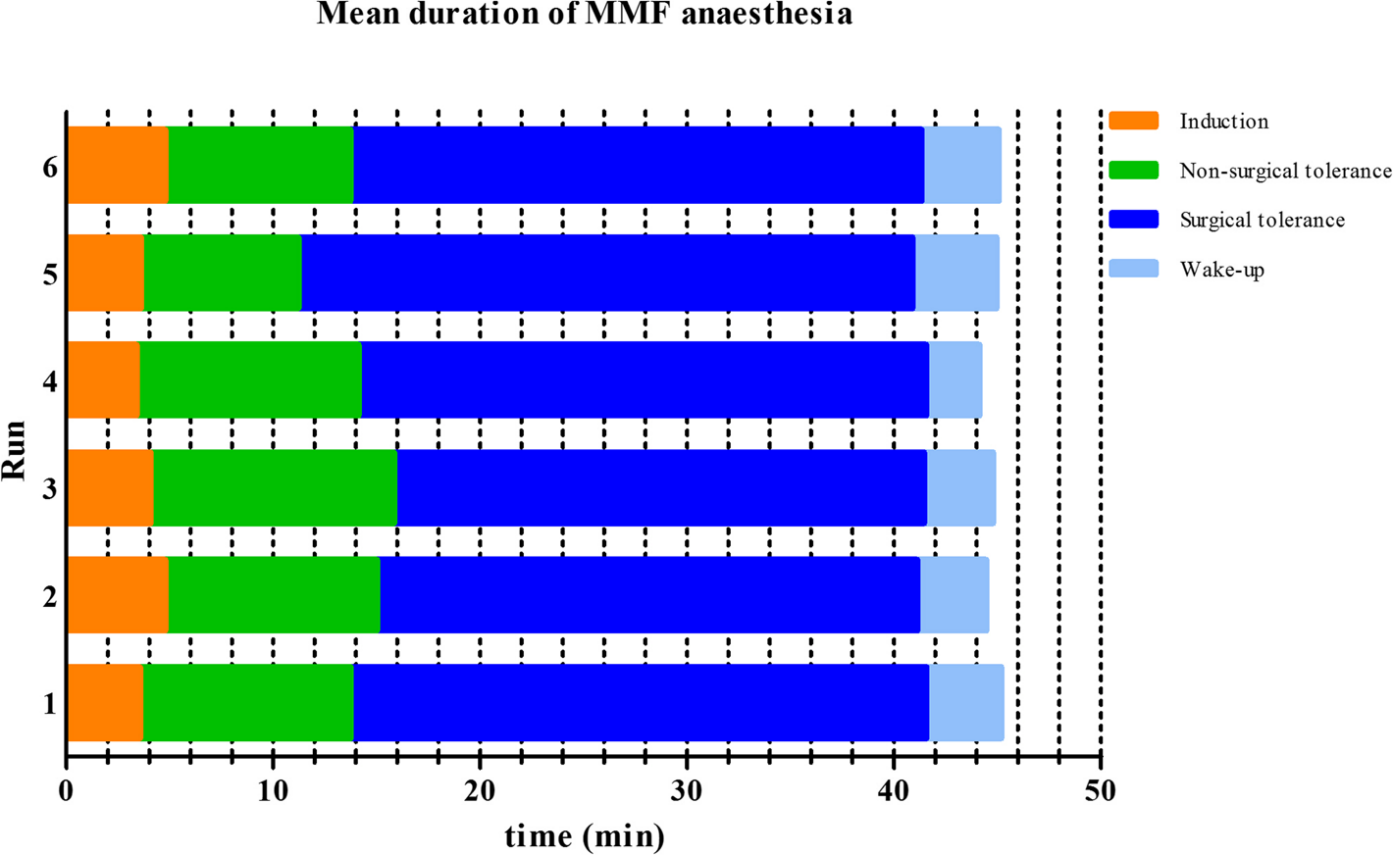
**Mean duration of MMF anaesthesia.** Duration of anaesthesia was divided in four different anaesthetic stages. A statistical significant difference (p value < 0.5) based on the ANOVA for the comparison of the anaesthetic stages of *run 2–6* with the anaesthetic stage of *run 1* was not observed.

The mean durations of the different anaesthetic stages of ISO, KX and MMF are depicted in Figures 1, 2 and 3, respectively. Significant changes were seen for ISO in *run 3*, when the stage of non-surgical tolerance and the time of surgical tolerance were compared with the same stages of *run 1* and in *run 5*, when the wake-up period was compared with the wake-up period of *run 1*. With KX significant differences of anaesthetic durations were observed. Induction times of *run 2* and *4* were significantly shorter than the induction time of *run 1* and times of non-surgical tolerance from *run 3–6* were significantly shorter when compared to *run 1*. For MMF, there were no significant differences observed in the duration of any anaesthetic stage between any of the runs.

### Heart rate, blood pressure values and body temperature

The mean values of HR, BP values and BT of ISO, KX and MMF anaesthesia with the statistical analysis are presented in Tables 1, 2 and 3, respectively. No significant differences were observed during baseline assessment, except in *run 3* and *run 4* for BT in ISO anaesthesia. The absolute differences of mean BT of run 3 and 4 to run 1 were only 0.22°C. These deviations were judged to be of no clinical relevance.

**Table 1:** **Isoflurane – mean values with standard deviation**

**Parameter**	**Run**	**Baseline**	**Induction**	**Non-surgical tolerance**	**Surgical tolerance**	**Wake-up**	**Recovery**
	**(No. of animals)**	**Mean ± SD**	**Mean ± SD**	**Mean ± SD**	**Mean ± SD**	**Mean ± SD**	**Mean ± SD**
**SAP** (mmHg)	1 (6)	121 ± 11.33	141 ± 13.09	133 ± 15.24	117 ± 7.76	123 ± 12.44	122 ± 11.41
	2 (6)	120 ± 11.55	140 ± 10.78	128 ± 16.02	113 ± 9.33	121 ± 10.11	122 ± 10.94
	3 (6)	121 ± 7.72	140 ± 11.81	130 ± 12.45	114 ± 8.71	123 ± 8.63	122 ± 8.45
	4 (6)	120 ± 13.04	139 ± 12.48	130 ± 12.84	111 ± 9.11	120 ± 12.45	119 ± 9.77
	5 (6)	115 ± 18.93	134 ± 16.08	124 ± 18.84	104 ± 16.35*	112 ± 20.32	116 ± 14.24
	6 (5)	120 ± 8.00	139 ± 9.83	129 ± 14.88	106 ± 12.51*	120 ± 16.26	121 ± 8.87
**DAP** (mmHg)	1 (6)	90 ± 7.73	106 ± 11.97	97 ± 12.48	85 ± 6.96	87 ± 11.22	92 ± 7.94
	2 (6)	89 ± 10.32	105 ± 10.06	92 ± 14.54	81 ± 7.68	86 ± 7.95	91 ± 8.58
	3 (6)	89 ± 6.26	103 ± 10.88	93 ± 9.52	82 ± 7.54	87 ± 5.22	91 ± 7.24
	4 (6)	88 ± 9.13	102 ± 10.85	93 ± 9.39	79 ± 6.44	85 ± 7.16	90 ± 8.45
	5 (6)	85 ± 11.05	101 ± 9.30	90 ± 10.33	75 ± 7.76*	80 ± 9.46	89 ± 8.00
	6 (5)	89 ± 7.55	103 ± 6.26	93 ± 10.85	75 ± 7.84*	84 ± 9.10	91 ± 7.64
**MAP** (mmHg)	1 (6)	100 ± 8.43	118 ± 11.99	109 ± 13.10	96 ± 6.72	99 ± 11.21	102 ± 8.72
	2 (6)	99 ± 10.48	116 ± 9.96	104 ± 14.79	92 ± 7.99	97 ± 8.36	101 ± 9.12
	3 (6)	100 ± 6.46	116 ± 10.94	105 ± 10.21	93 ± 7.41	99 ± 5.71	102 ± 7.31
	4 (6)	99 ± 10.20	115 ± 11.11	105 ± 10.28	90 ± 6.93	97 ± 8.68	100 ± 8.79
	5 (6)	95 ± 13.43	112 ± 11.17	102 ± 12.93	85 ± 10.42*	90 ± 13.00	98 ± 9.86
	6 (5)	100 ± 7.28	115 ± 7.01	105 ± 11.92	85 ± 9.16*	96 ± 11.40	101 ± 7.69
**PP** (mmHg)	1 (6)	32 ± 7.21	35 ± 6.27	36 ± 6.57	32 ± 5.69	35 ± 6.65	30 ± 6.49
	2 (6)	31 ± 5.01	35 ± 5.63	35 ± 5.84	32 ± 4.55	35 ± 5.33	30 ± 5.14
	3 (6)	32 ± 4.40	37 ± 5.07*	37 ± 5.89	32 ± 6.14	36 ± 6.83	31 ± 4.86
	4 (6)	31 ± 6.06	37 ± 5.58*	37 ± 5.99	32 ± 5.72	35 ± 6.88	30 ± 3.14
	5 (6)	29 ± 9.60	33 ± 9.24	34 ± 10.02	29 ± 9.63	32 ± 11.29	27 ± 7.66
	6 (5)	31 ± 5.37	36 ± 6.44	36 ± 6.82	31 ± 6.47	35 ± 7.82	29 ± 5.18
**HR** (bpm)	1 (6)	307 ± 27.11	417 ± 37.53	385 ± 22.02	389 ± 20.42	420 ± 22.31	297 ± 16.06
	2 (6)	291 ± 16.65	392 ± 33.78	364 ± 18.80*	379 ± 16.73	409 ± 19.53*	297 ± 16.66
	3 (6)	295 ± 12.59	393 ± 26.09*	365 ± 15.65*	377 ± 20.73*	411 ± 20.94*	295 ± 11.67
	4 (6)	297 ± 25.77	401 ± 28.61	366 ± 20.41*	373 ± 21.72*	407 ± 19.90*	284 ± 12.68*
	5 (6)	288 ± 17.14	387 ± 29.30*	358 ± 18.14*	366 ± 16.16*	406 ± 16.86*	291 ± 9.42
	6 (5)	298 ± 18.58	386 ± 41.82	352 ± 24.35*	367 ± 7.26*	412 ± 18.68*	289 ± 13.51
**BT** (°C)	1 (6)	37.62 ± 0.49	37.52 ± 0.50	37.48 ± 0.52	36.50 ± 0.45	36.11 ± 0.52	37.26 ± 0.21
	2 (6)	37.46 ± 0.43	37.43 ± 0.34	37.35 ± 0.33	36.19 ± 0.37	35.71 ± 0.41	37.25 ± 0.16
	3 (6)	37.40 ± 0.37*	37.30 ± 0.28	37.20 ± 0.35	36.22 ± 0.35	35.82 ± 0.47	37.19 ± 0.32
	4 (6)	37.40 ± 0.37*	37.39 ± 0.22	37.29 ± 0.24	36.32 ± 0.17	35.90 ± 0.37	37.25 ± 0.18
	5 (6)	37.46 ± 0.33	37.27 ± 0.29	37.17 ± 0.38	36.16 ± 0.27	35.72 ± 0.30	37.13 ± 0.32
	6 (5)	37.56 ± 0.38	37.55 ± 0.25	37.45 ± 0.26	36.20 ± 0.52*	35.71 ± 0.60*	37.20 ± 0.12

*Statistical significant difference (p value ≤ 0.05) based on the ANCOVA with baseline as covariate for the comparison of anaesthetic stages between *run 2–6* versus *run 1*.

**Table 2:** **Ketamine-Xylazine– mean values with standard deviation**

**Parameter**	**Run**	**Baseline**	**Induction**	**Non-surgical tolerance**	**Recovery**
	**(No. of animals)**	**Mean ± SD**	**Mean ± SD**	**Mean ± SD**	**Mean ± SD**
**SAP** (mmHg)	1 (6)	116 ± 11.27	130 ± 12.81	96 ± 12.38	104 ± 9.16
	2 (6)	118 ± 11.85	133 ± 13.38	97 ± 7.07	106 ± 8.86
	3 (6)	114 ± 7.93	134 ± 11.38	96 ± 10.78	106 ± 6.18
	4 (6)	116 ± 10.03	138 ± 10.86*	95 ± 9.36	105 ± 6.09
	5 (6)	115 ± 9.65	133 ± 6.83	100 ± 9.89	103 ± 5.40
	6 (5)	115 ± 6.14	138 ± 6.62	102 ± 6.18	105 ± 4.85
**DAP** (mmHg)	1 (6)	85 ± 9.85	96 ± 9.39	68 ± 10.01	76 ± 6.38
	2 (6)	88 ± 10.40	96 ± 13.27	69 ± 7.57	77 ± 8.68
	3 (6)	86 ± 10.10	103 ± 7.13	69 ± 9.81	78 ± 6.66
	4 (6)	87 ± 8.15	103 ± 8.76	69 ± 7.13	75 ± 2.87
	5 (6)	86 ± 7.04	99 ± 7.09	72 ± 9.14	75 ± 2.57
	6 (5)	84 ± 5.83	102 ± 6.44	71 ± 5.24*	74 ± 1.39
**MAP** (mmHg)	1 (6)	95 ± 10.13	108 ± 10.35	77 ± 10.65	85 ± 7.08
	2 (6)	98 ± 10.40	108 ± 12.93	79 ± 6.68	87 ± 8.34
	3 (6)	95 ± 8.86	113 ± 8.12	78 ± 9.29	87 ± 5.76
	4 (6)	96 ± 8.40	115 ± 9.05	78 ± 6.92	85 ± 3.64
	5 (6)	96 ± 7.24	110 ± 6.16	81 ± 8.42	84 ± 2.20
	6 (5)	95 ± 5.50	114 ± 6.35	81 ± 5.07	85 ± 2.39
**PP** (mmHg)	1 (6)	31 ± 4.42	34 ± 5.40	28 ± 4.52	28 ± 4.74
	2 (6)	30 ± 6.95	36 ± 6.67*	28 ± 6.76	29 ± 5.51
	3 (6)	28 ± 6.89	32 ± 7.08	27 ± 8.65	27 ± 6.39
	4 (6)	30 ± 5.73	35 ± 6.20*	27 ± 8.29	30 ± 4.57*
	5 (6)	29 ± 7.22	33 ± 7.09	28 ± 8.86	28 ± 6.47
	6 (5)	31 ± 4.71	36 ± 2.97	31 ± 4.91	31 ± 3.94*
**HR** (bpm)	1 (6)	283 ± 36.14	354 ± 38.00	273 ± 23.56	288 ± 19.35
	2 (6)	284 ± 20.10	375 ± 45.16	281 ± 20.12	292 ± 12.37
	3 (6)	278 ± 14.31	359 ± 33.95	273 ± 19.86	296 ± 21.69
	4 (6)	281 ± 22.22	340 ± 28.56	275 ± 35.47	290 ± 27.95
	5 (6)	285 ± 20.98	362 ± 31.88	275 ± 29.33	292 ± 13.50
	6 (5)	282 ± 21.89	362 ± 10.24	269 ± 21.50	277 ± 18.54
**BT** (°C)	1 (6)	37.39 ± 0.59	37.34 ± 0.63	36.96 ± 0.48	37.57 ± 0.47
	2 (6)	37.49 ± 0.27	37.44 ± 0.22	37.34 ± 0.32*	37.79 ± 0.32
	3 (6)	37.42 ± 0.19	37.27 ± 0.24	37.18 ± 0.37	37.72 ± 0.31
	4 (6)	37.59 ± 0.23	37.40 ± 0.26	37.35 ± 0.45*	37.54 ± 0.53
	5 (6)	37.56 ± 0.37	37.55 ± 0.39	37.46 ± 0.45*	37.79 ± 0.32
	6 (5)	37.73 ± 0.48	37.58 ± 0.58	37.43 ± 0.36*	37.79 ± 0.08

*Statistical significant difference (p value ≤ 0.05) based on the ANCOVA with baseline as covariate for the comparison of anaesthetic stages between *run 2–6* versus *run 1*.

**Table 3:** **Medetomidine-midazolam-fentanyl – mean values with standard deviation**

**Parameter**	**Run**	**Baseline**	**Induction**	**Non-surgical tolerance**	**Surgical tolerance**	**Wake-up**	**Recovery**
	**(No. of animals)**	**Mean ± SD**	**Mean ± SD**	**Mean ± SD**	**Mean ± SD**	**Mean ± SD**	**Mean ± SD**
**SAP** (mmHg)	1 (6)	117 ± 5.42	158 ± 19.28	160 ± 21.24	157 ± 23.24	101 ± 15.20	113 ± 9.43
	2 (6)	111 ± 7.34	144 ± 19.22*	151 ± 24.77	151 ± 25.47	105 ± 15.15	113 ± 7.08
	3 (6)	112 ± 9.32	153 ± 18.24	152 ± 23.21	147 ± 24.90*	101 ± 13.70	115 ± 7.90
	4 (5)	116 ± 8.18	149 ± 19.24	157 ± 28.86	146 ± 20.77	102 ± 11.90	114 ± 11.13
	5 (6)	115 ± 6.49	153 ± 19.81	154 ± 21.44	146 ± 25.10*	103 ± 10.03	117 ± 9.90
	6 (5)	117 ± 11.54	150 ± 17.01	146 ± 14.86*	148 ± 26.76	109 ± 17.16*	121 ± 11.21*
**DAP** (mmHg)	1 (6)	89 ± 4.40	120 ± 15.38	112 ± 7.78	107 ± 12.31	76 ± 13.69	90 ± 11.00
	2 (6)	84 ± 7.51	112 ± 10.63	106 ± 12.00	103 ± 12.22	81 ± 10.84	90 ± 6.40
	3 (6)	84 ± 5.49	117 ± 12.26	108 ± 9.00	103 ± 11.24	77 ± 11.00	91 ± 9.09
	4 (5)	89 ± 13.66	114 ± 6.48	111 ± 15.30	101 ± 6.64	76 ± 4.93	91 ± 13.87
	5 (6)	87 ± 5.85	116 ± 9.81	112 ± 8.85	103 ± 9.61	78 ± 8.78	92 ± 10.43
	6 (5)	89 ± 12.07	117 ± 10.93	110 ± 7.56	106 ± 10.62	84 ± 12.95	97 ± 11.12*
**MAP** (mmHg)	1 (6)	98 ± 2.90	132 ± 15.27	128 ± 10.61	123 ± 14.23	84 ± 13.90	98 ± 10.09
	2 (6)	93 ± 6.90	123 ± 12.88*	121 ± 15.41	119 ± 15.59	89 ± 12.11	98 ± 6.27
	3 (6)	93 ± 5.91	129 ± 12.90	123 ± 12.68	118 ± 14.39	85 ± 11.55	99 ± 8.38
	4 (5)	98 ± 11.61	126 ± 9.62	126 ± 19.01	116 ± 10.48	84 ± 6.59	99 ± 12.78
	5 (6)	96 ± 5.27	128 ± 12.79	126 ± 12.53	117 ± 14.16	86 ± 8.81	101 ± 9.98
	6 (5)	98 ± 11.39	128 ± 12.03	122 ± 8.47	120 ± 15.02	93 ± 14.07*	105 ± 10.66*
**PP** (mmHg)	1 (6)	28 ± 8.01	38 ± 14.80	48 ± 18.77	51 ± 18.78	25 ± 6.32	23 ± 6.17
	2 (6)	27 ± 5.94	33 ± 12.11	45 ± 16.81	48 ± 18.10	24 ± 6.08	23 ± 4.64
	3 (6)	28 ± 7.98	36 ± 14.18	43 ± 18.11	44 ± 19.42*	24 ± 6.67	24 ± 5.03
	4 (5)	27 ± 7.33	35 ± 16.25	46 ± 18.03	45 ± 16.88	27 ± 9.47	23 ± 5.29
	5 (6)	28 ± 6.38	37 ± 11.88	42 ± 14.75*	43 ± 17.89*	25 ± 5.78	24 ± 5.08
	6 (5)	28 ± 7.29	33 ± 11.90*	36 ± 13.39*	42 ± 19.92*	25 ± 7.30	24 ± 6.96
**HR** (bpm)	1 (6)	289 ± 23.42	285 ± 37.92	211 ± 16.99	197 ± 19.63	249 ± 19.49	275 ± 15.28
	2 (6)	274 ± 14.20	285 ± 45.73	214 ± 14.44	195 ± 15.42	255 ± 23.43	276 ± 15.52
	3 (6)	277 ± 19.13	298 ± 26.01	215 ± 17.93	194 ± 16.58	247 ± 11.99	274 ± 19.53
	4 (5)	282 ± 30.50	290 ± 26.66	214 ± 15.00	193 ± 15.97	251 ± 15.56	278 ± 20.03
	5 (6)	276 ± 18.01	290 ± 39.66	212 ± 15.61	193 ± 13.88	257 ± 30.82	282 ± 24.58*
	6 (5)	271 ± 15.55	298 ± 42.95*	215 ± 13.24*	191 ± 9.46	272 ± 43.96*	279 ± 26.52*
**BT** (°C)	1 (6)	37.50 ± 0.21	37.18 ± 0.16	36.88 ± 0.25	36.09 ± 0.17	35.67 ± 0.20	37.17 ± 0.58
	2 (6)	37.48 ± 0.34	37.44 ± 0.31	37.05 ± 0.34	36.11 ± 0.25	35.65 ± 0.17	37.14 ± 0.48
	3 (6)	37.27 ± 0.29	37.17 ± 0.36	36.86 ± 0.25	36.07 ± 0.26	35.66 ± 0.36	37.14 ± 0.36
	4 (5)	37.58 ± 0.31	37.50 ± 0.37	37.24 ± 0.36*	36.28 ± 0.24	35.78 ± 0.25	37.32 ± 0.26
	5 (6)	37.46 ± 0.19	37.13 ± 0.39	36.94 ± 0.42	36.08 ± 0.36	35.63 ± 0.28	37.23 ± 0.31
	6 (5)	37.28 ± 0.28	37.18 ± 0.15	36.90 ± 0.16	35.96 ± 0.35	35.53 ± 0.42	37.38 ± 0.29

*Statistical significant difference (p value ≤ 0.05) based on the ANCOVA with baseline as covariate for the comparison of anaesthetic stages between *run 2–6* versus *run 1*.

**ISO**: During ISO the most significant alterations were seen in HR. HR in *run 3* and *5* during induction time, in *run 2–6* during time of non-surgical tolerance, in *run 3–6* during time of surgical tolerance and in *run 2–6* during the wake-up period were significantly lower compared to the HR of *run 1* . For induction time, time of non-surgical tolerance and time of surgical tolerance we noted a continuous decrease of HR with repeated anaesthesia. For SAP, DAP and MAP significantly lower values were observed during time of surgical tolerance if *run 5* and *6* were compared with *run 1*. PP was significantly higher during induction time in *run 3* and *4* compared to *run 1* and BT was significantly lower in *run 6* during time of surgical tolerance and during wake-up period compared to *run 1*.

**KX**: With KX significant differences of BP values compared to *run 1* were only observed once during induction time (SAP in *run 4*) and during the time of non-surgical tolerance (DAP in *run 6*). PP was significantly higher during induction time in *run 2* and *4* and during recovery period in *run 4* and *6* if compared to *run 1*. During time of non-surgical tolerance BT was significantly higher in *run 2*, *4*, *5* and *6*, when compared to *run 1*.

**MMF**: Significant differences of BP values with MMF could be observed during the induction time. SAP and MAP were significantly lower in *run 2* compared to *run 1.* SAP was also significantly lower during time of non-surgical tolerance in *run 6* and during time of surgical tolerance in *run 3* and *5*. During the wake-up period, SAP and MAP showed significantly higher values in *run 6* and during the recovery period SAP, DAP and MAP were significantly higher in *run 6* compared to *run 1*. PP showed significantly lower values during induction time in *run 6*, during time of non-surgical tolerance in *run 5* and *6* and during time of surgical tolerance in *run 3*, *5* and *6*, when each was compared to *run 1* of its respective anaesthetic stage. A significantly higher HR could be observed in *run 6*, if compared to the *1st run*, during induction time, time of non-surgical tolerance and wake-up period. During the recovery period HR was significantly higher in *run 5* and *6*, if compared to *run 1*. BT was significantly higher in *run 4* compared to *run 1* during time of non-surgical tolerance.

### Body weight

**Figure 4:**
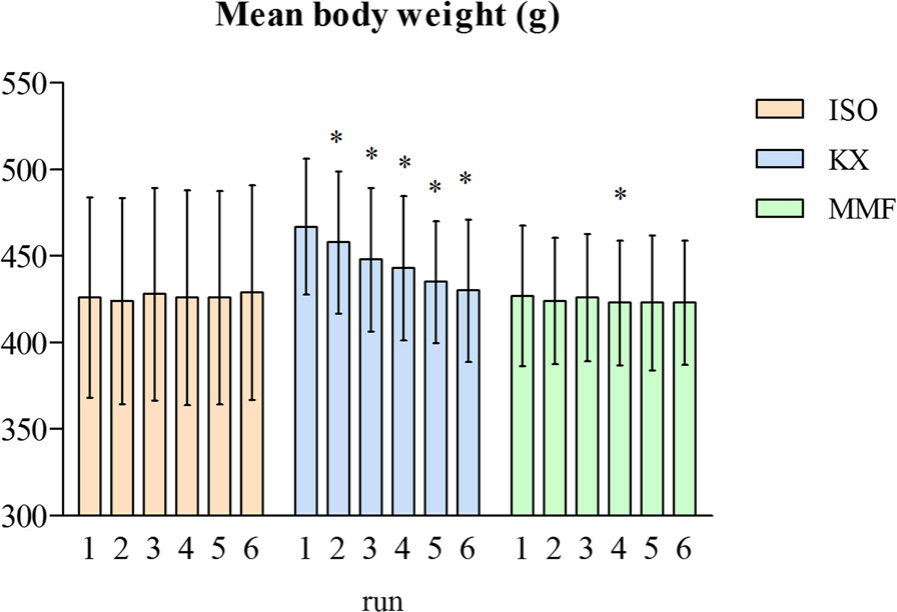
**Mean body weight.** *Statistical significant difference (p < 0.05) based on the ANOVA for the comparison of body weight between *run 2–6* versus *run 1*.

Mean BW including the statistical analysis is depicted in Figure 4. No significant differences of BW could be observed during repeatedly performed anaesthesia with ISO. With KX a continuous decrease of BW was seen with an increased number of performed anaesthesias. The reduction of BW was significant from *run 2* to *6*, if compared with *run 1*. A reduction of BW was significant once for MMF in *run 4*, if compared to BW of *run 1*.

### Tissue reaction to injections

Clinical effects on the hind legs were seen in four rats anaesthetized with KX (a total of six rats were repeatedly anaesthetized with KX). In two rats we observed tissue necrosis on the right hind leg. Both hind legs were affected with necrotic lesions in two other rats. The time of the first occurrence of the tissue necrosis differed between the four rats. The earliest occurrence of the necrosis was one day after the second anaesthesia in two rats and the other necrosis occurred after the fourth and fifth run with KX.

## Discussion

This study investigated if repeated anaesthesia with ISO, KX or MMF alter their influence on HR or BP values, BT and BW of male Wistar rats. To our knowledge, it was the first time that these parameters, together with the duration of anaesthetic intervals were evaluated during repeatedly performed anaesthesia using telemetric data assessment.

### Effects on duration of anaesthesia

**ISO**: The significant differences in duration of anaesthetic stages we observed with ISO occurred only sporadically, once in the time of non-surgical tolerance (*run 3*), once in the time of surgical tolerance (*run 3*) and once in the wake-up period (*run 5*). It must be kept in mind, however, that each rat needed a slightly different concentration of ISO (between 2–3 Vol%) to reach a defined stage of anaesthesia. This resulted in different times to adjust the ISO. Furthermore, the total length of surgical tolerance depended on the duration of the induction time and time of non-surgical tolerance, because we terminated ISO delivery after 40 minutes. Thus, it seemed inappropriate to interpret the duration of non-surgical and surgical tolerance. Finally, since the absolute differences in the durations were less than three minutes, we do not classify these differences, despite statistical significant, as being clinically relevant.

**KX**: With KX there was tendency for the sleeping time to decrease from *run 1* to *run 6*. These findings are in accordance with other studies evaluating the sleeping time during ketamine or ketamine-medetomidine anaesthesia in which the authors suggested an increased induction of metabolizing enzymes, which resulted in an increased clearance of the anaesthetics [17-21]. In one of these studies the authors stated that the hypnotic effect was caused by a specified plasma level of ketamine in the brain and that the duration of anaesthesia depends on the speed of tissue redistribution and metabolization [18]. Furthermore, a faster ketamine metabolism during anaesthesia has been demonstrated after a daily low-dose ketamine pretreatment over ten days, which is consistent with there being an increase in metabolizing enzymes [17,20]. We suggest that similar mechanisms may have caused the decrease of sleeping time after repeatedly performed KX anaesthesia in the present study. A relationship between the continuous reduction of sleeping time and the failure to reach surgical tolerance was not observed, because some rats failed to reach this stage in almost all *runs* (except in *run 4*) and therefore this was not associated with the increased number of anaesthesias. The inconsistence of ketamine-xylazine to produce anaesthesia of sufficient depth has been reported in previous studies [22,23]. This effect has also been observed with the combinations of ketamine-medetomidine or ketamine-dexmedetomidine [24]. Since it was also suggested that the risk of anaesthetic mortality may increase with the application of higher dosages, we chose not to increase the ketamine-xylazine dosage in this study [23].

**MMF**: With MMF no significant differences in the duration of the anaesthetic stages were observed with repeated anaesthesia. In contrast to KX, MMF was antagonized after 40 minutes. Therefore, it was not possible to detect a change in whole sleeping time even if there had been an increase of metabolizing enzymes. It would be possible if rats became conscious before antagonization, but this did not occur in this study. As with ISO we were not able to evaluate alterations of the time of surgical tolerance, but for induction time, time of non-surgical tolerance and wake-up period, repeated MMF anaesthesia had no effect.

### Effects on heart rate, arterial blood pressure and body temperature

**Figure 5:**
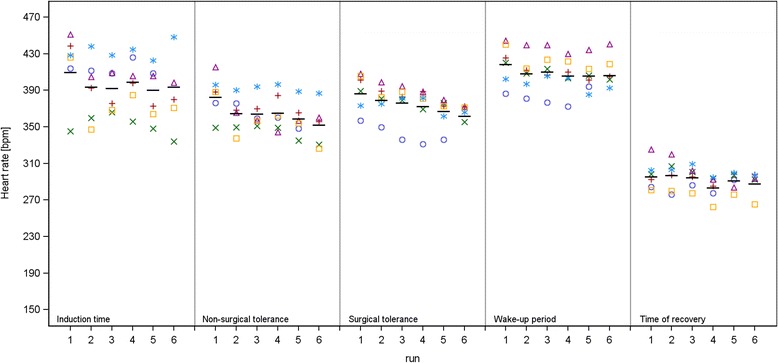
**Isoflurane – Heart rate.** Individual values per animal (colour highlighted symbols) and adjusted mean (−) by anaesthetic interval.

**ISO**: With repeated anaesthesia using ISO, there was a decrease in arterial BP. The decrease was significant from *run 5–6* in comparison to run 1 during the time of surgical tolerance. Significant lower HR compared to *run 1* could also be observed during the induction time, time of non-surgical and surgical tolerance and wake-up period in almost all subsequent *runs* (*run 2–6*). The HR decrease is depicted in Figure 5. This was an unexpected effect, because with decreasing BP values one would usually expect a compensatory increase of HR through baroreceptor-mediated effects. It seemed that the depressant effect of ISO on BP became more pronounced with an increased number of performed anaesthesias, but the ability to compensate for this BP decrease with an increased HR also diminished. Repeated ISO anaesthesia did not have any effect on myocardial, anatomical or histological endpoints in previous studies [25,26]. A further study noticed no significant QT prolongation in the ECG, no myocardial repolarization abnormalities or any evidence for left ventricular enlargement after repeated anaesthesia with ISO [27]. Taken together, a histopathological explanation for the sequential depression of HR and BP appears not to be the case. Another aspect could be that the ISO concentration was adapted to the needs of each individual rat (2–3 Vol%). This variable, not standardized ISO concentration could have had an influence on the measured parameters. The decreases in BP and HR we observed during six anaesthesias were not severe enough to require intervention and are not interpreted to be indicative of acute heart failure. Whether or not the extent of the decrease in HR and BP would be greater if more repetitions of ISO anaesthesia were performed is not known. The significant increase of PP observed during induction time in *run 3* and *4* were not associated with significant differences of the absolute BP values in the same *runs* and these two PP values were still well within the physiological range [5]. Therefore, we concluded that the observed increase of PP had no clinical relevance. In *run 6* BT was significantly lower compared to *run 1* in time of surgical tolerance and wake-up period. A possible explanation could be the lower BP values in *run 6*, due to the vasodilative effects of ISO, leading to a greater heat loss during anaesthesia [6].

**KX**: The significant changes of BP parameters during repeated KX anaesthesia occurred sporadically and did not correlate with the different anaesthetic stages. In our previous study, which evaluated the effect of a single KX anaesthesia, we observed similar BP values compared to those indicated to be different compared to *run 1*, suggesting that changes were without clinical relevance, since all values were within normal, physiological ranges [5,9,28]. During the time of non-surgical tolerance BT showed a tendency to increase and was significantly different to *run 1* in *run 2*, *4*, *5* and *6*. As mentioned above, the duration of this anaesthetic stage decreased progressively from *run 1–6*. It is well known that anaesthetics influence the thermoregulatory control and that especially small animals, like rodents, due to their large surface area to body weight ratio, lose heat rapidly and may become hypothermic during prolonged anaesthesia [6,8]. With the duration of anaesthesia becoming shorter from *run 1–6*, there was less time for the rats to lose body heat and therefore, BT was comparatively higher during the time of non-surgical tolerance. One animal receiving KX died during its sixth anaesthesia. After injection of the anaesthetics the rat immediately lost its righting reflex. HR and BP decreased markedly and the animal stopped breathing. Different mortality rates have been reported previously for ketamine-xylazine, but without post-mortem data we could not exclude any other factors (e.g. latent infections) that may have contributed to the rat’s death [23,29]. Many investigators now use ketamine combined with medetomidine or dexmedetomidine rather than xylazine. Anaesthesia of similar quality and depth was reported for the combination of ketamine-xylazine and ketamine-dexmedetomidine in rats [30]. Another study evaluating the effects of repeated ketamine-medetomidine anaesthesia reported that anaesthesia was not associated with anaesthetic mortalitiy, but in combination with buprenorphine they observed an increased risk of anaesthetic related death [31].

**Figure 6:**
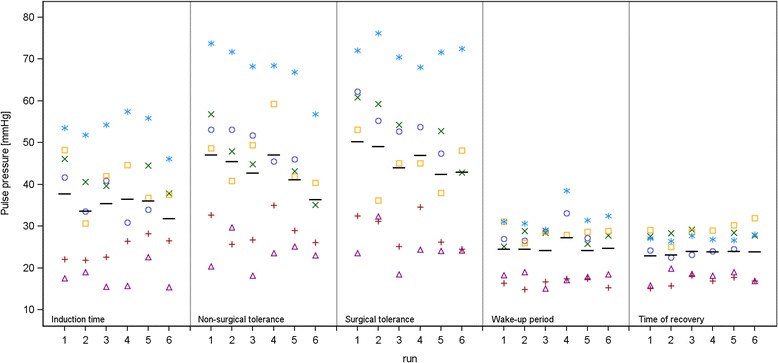
**Medetomidine-midazolam-fentanyl – Pulse pressure.** Individual values per animal (colour highlighted symbols) and adjusted mean (−) by anaesthetic interval.

**MMF**: From induction time to the time of surgical tolerance a slight decrease of BP values, especially PP, could be observed from *run 1–6*, whereas during wake-up and recovery periods BP parameters slightly increased with a significant difference only observed in *run 6*. HR tended to increase from *run 1–6* but not during the time of surgical tolerance. The increased HR was only significant, however, in *run 6*. In a previous report on the effects of repeated MMF anaesthesia in rats, the authors observed only a slight decrease of a vitality index (escape reaction, aggressiveness, speed of movements and paresis were classified and scored) [32]. They did not observe significant differences of HR and BT when performing ten anaesthesias within three months [32]. In our previous study we observed significant increases of all BP values, with PP almost doubled compared to its baseline value and HR was significantly decreased during MMF anaesthesia [9]. As also seen in this study, a rapid and short-lasting decrease of BP values occurred after antagonism. We noted that the BP values of *run 1* were already lower compared to the values of this single MMF anaesthesia, although we had expected approximately similar values at least during the *1st run* of MMF anaesthesia. Furthermore, we observed a much larger variability with the repeated anaesthesia compared to the variability seen with the same anaesthetic stages in the previous study. Baseline values of these two studies were comparable, therefore, the cardiovascular condition of the rats and the quality of the telemetric signal were comparable during time of anaesthetic induction. We conclude, therefore, that the effect of MMF anaesthesia on haemodynamic parameters was indeed more variable as in the previous study. An example of the great interindividual difference of PP values is depicted in Figure 6. We noticed that the anaesthetic profiles were most similar to the previous study with the youngest animal in the group, whereas lowest BP values during MMF anaesthesia were mostly seen in the older animals. The differences of *run 1* compared to the values of the previous study could be related to the different ages of the rats (maximum of five months age difference). It is known that metabolism and mechanisms of the autonomic nervous system and cardiovascular system undergo age-related changes [6,33]. Effects of age have been described for other anaesthetic regimes, but have not yet been reported for haemodynamic parameters during MMF treatment [34,35]. BT was not affected substantially by repeated anaesthesia, with only one significant difference during time of-non surgical tolerance in *run 4*. If we compared the BT of the repeated anaesthesia with the BT values of the previous study, there was a difference of almost one degree, especially during time of surgical tolerance and wake-up period, although the same conditions were provided to maintain heat throughout the measurements [9]. Baseline values of BT and recovery values of these two studies were comparable. Therefore, the influence of MMF anaesthesia on BT in the two studies appears to be different. Fentanyl and midazolam have only little effect on BP values, but medetomidine is known to cause a vasoconstriction in the peripheral circulation and loss of body heat depending, among other things, on the perfusion of the periphery [5,6]. Medetomidine might have caused a less intensive vasoconstriction which resulted in lower BP values in this study compared to the values of the previous study. An effect of age or BW could also be responsible, but BT with ISO or KX anaesthesia was comparable between the two studies and there were the same differences in age and BW. Therefore, an age- or BW-related effect seems unlikely. Nevertheless, the effect on BT was consistent within the three weeks of performing six MMF anaesthesias.

### Effects on body weight

BW did not alter significantly during the three weeks of repeated ISO and MMF anaesthesia, except at one time point with MMF (*run 4*). This difference was, however, a BW reduction of less than 1% compared to the BW in *run 1*. Hence, it is not of clinical relevance, since BW remained unaffected in the following *runs* using MMF anaesthesia. A continuous decrease of BW was observed with KX, however. The reduction of mean BW from *run 1–6* was nearly 8%. This could be due to the long sleeping time and recovery period, such that the rats ate and drank less. Another negative effect on food and water uptake could be the disturbing influence of ketamine on the circadian rhythm of the animal [36,37]. The seriousness of a BW loss of 8% depends on the initial body weight of the animal. Decreases in body weight in the range of 15-30%, alone or in combination with a poor general condition, have been used in the past to indicate the necessity for a humane endpoint of a test animal [38,39]. We did not reach a BW loss of this extent after performing six anaesthesias within three weeks, but it could be more pronounced if performing more than *6 runs* or reducing the time between two anaesthesias. Furthermore, one has to consider the additional influence of experimental procedures (surgery, drug application, etc.) on BW.

### Effects of administration route

No obvious detrimental effects could be observed with repeated ISO inhalation or i.m. administration of MMF, but with KX, we often noticed visible tissue necrosis at the injection site. A reason could be the administered volume of anaesthetics. The injected volumes of KX and MMF in this study appear higher than volumes recommended for intramuscular injection in rats in some guidelines [40]. However, recommendations are typically given in volume per animal (ml) as opposed to volume per kg body weight. The rats used in this study had a mean body weight of 434 ± 48 g and thus were substantially heavier than commonly used laboratory rats. We chose not to administer the anaesthetics intraperitoneally, because of the presence of the telemetry device in the abdominal cavity and the risk to damage the cables during injection. Additionally, peritonitis as a result of intraperitoneal injection of irritants would be associated with more complications in rats implanted with a telemetry device. Furthermore, an intraperitoneal injection has a risk to be misdirected into the gastrointestinal tract or the urinary bladder [6,41,42]. This depends on how familiar one is with the technique and differed between personnel. The variable failure rate may have serious consequences for analysis and data interpretation [43,44]. We suggest that the acidic formulation of ketamine, and not the injected volume, caused these necrotic lesions, because the injected volume of KX at each injection site was comparable with that of MMF. Of course, we cannot exclude the possibility, that also MMF caused necrotic lesions to a lesser extent, which were not obvious visible during our daily clinical monitoring. Histological examinations, which were not carried out in this study, are required to clarify the tissue response after an intramuscular injection of MMF. We treated the wounds at the injection sites daily with an ointment (Pana Veyxal® Salbe, Veyx-Pharma GmbH, Schwarzenborn, Germany) to accelerate the healing process, but with an increased number of performed KX anaesthesias, the tissue necrosis became worse. The size of the necrosis did not become too large, so that an injection of subsequent KX administrations in healthy tissue was still possible. The absorption of drugs injected in inflamed muscle tissue is different due to an altered tissue pH and an increased vascularization [45,46]. Duration and depth of anaesthesia could be influenced as well as HR, BP and BW. Therefore, a comparison of anaesthesias injected in healthy and inflamed tissue is not advisable.

Altogether, with regard to a low-stress administration and smooth and rapid induction of anaesthesia, ISO offers a clear advantage compared to the injectable anaesthetics. Additionally, it cannot be denied that ISO is only minimally metabolized compared to the other anaesthetics, easily adjustable to the individual needs of each animal and simple to handle and to prolong the duration of anaesthesia if needed. These advantageous aspects of ISO have to be taken into consideration for the repeated use, unless the experimental procedure would not allow an inhalational anaesthetic regimen.

## Conclusion

Our study evaluated the effect of repeated anaesthesia with ISO, KX or MMF on HR, SAP, DAP, MAP, PP, BT, duration of anaesthetic intervals and BW in male Wistar rats.

With the decrease of HR and BP during the six repetitive ISO anaesthesia, the use of ISO has to be considered if a change of these parameters is not acceptable for a given study design. Otherwise, ISO is well-suited for serial use, since it is possible to produce anaesthesia with comparable duration and depth and it has some advantageous aspects compared to the injectable regimes. With the inability to consistently produce a surgical plane of anaesthesia and the reduction of sleeping time and BW, as well as the frequent production of a local tissue necrosis at the injection site, KX has some distinct disadvantages. Although HR, BP values and BT remained unaffected after six KX anaesthesias, the other results of this study may be considered significant contraindications for the repeated use of KX. With only mild changes of BP values with repeated anaesthesia, MMF seems suitable for serial use, unless the high BP and the low HR during the surgical plane of anaesthesia are undesirable.

## Abbreviations

AFN: Atipamezole-flumazenil-naloxone
BP: Blood pressure
bpm: Beats per minute
BT: Body temperature
BW: Body weight
DAP: Diastolic arterial pressure
DSI™: Data Science International™
fig.: Figure
HR: Heart rate
i.m.: Intramuscular
ISO: Isoflurane
KX: Ketamine-xylazine
MAP: Mean arterial pressure
min: Minute
MMF: Medetomidine-midazolam-fentanyl
PP: Pulse pressure
SAP: Systolic arterial pressure
s.c.: Subcutaneous
SD: Standard deviation
